# Attitudes and Perceptions of Early-Career Psychiatrists Towards Electroconvulsive Therapy (ECT) in Poland: A Call for Enhanced Training and Guidelines

**DOI:** 10.1192/j.eurpsy.2024.1468

**Published:** 2024-08-27

**Authors:** M. E. Gołębiewska, A. Wilkowska, W. J. Cubała, M. Pinto da Costa

**Affiliations:** ^1^Department of Developmental, Psychotic, and Geriatric Psychiatry; ^2^Department of Psychiatry, Medical University of Gdansk, Gdansk, Poland; ^3^Institute of Psychiatry, Psychology & Neuroscience, King’s College London, London, United Kingdom; ^4^Institute of Biomedical Sciences Abel Salazar, University of Porto, Porto, Portugal

## Abstract

**Introduction:**

In Poland, the therapeutic modality of Electroconvulsive Therapy (ECT) boasts a history spanning over seven decades. Despite its documented therapeutic efficacy and safety profile, its integration into clinical practice remains suboptimal. Recent data elucidates a marked paucity in the utilization rate of ECT in Poland. Therefore, it is imperative to discern the barriers impeding its broader adoption of this potentially life-saving treatment.

**Objectives:**

The aim of this study is to investigate the attitude of early career psychiatrists towards ECT and its place in clinical practice in Poland.

**Methods:**

A web-based, anonymous survey was conducted, targeting early career psychiatrists in Poland. The questionnaire, part of an international study, consisted of 36 multiple-choice and Likert scale questions.

**Results:**

The majority of respondents emphasised the importance of further educational opportunities related to ECT, seeing it as a safe, effective, and possibly lifesaving procedure. Most of them benefited from ECT training during their residency, however less than a half had the opportunity to administer ECT themselves. They exhibited an interest to introduce ECT into their therapeutic repertoire, depending on the provision of requisite financial and infrastructural support.

**Conclusions:**

There is a palpable eagerness among early career psychiatrists in Poland to enhance their proficiency in ECT. A robust curriculum, encompassing both theoretical discourse and hands-on ECT training, is paramount for all psychiatry trainees. Concurrently, there is a pressing need to formulate national ECT guidelines within Poland, which could potentially ameliorate apprehensions surrounding this procedure.

**Disclosure of Interest:**

None Declared

